# Review of Water-Assisted Crystallization for TiO_2_ Nanotubes

**DOI:** 10.1007/s40820-018-0230-4

**Published:** 2018-11-15

**Authors:** Xiaoyi Wang, Dainan Zhang, Quanjun Xiang, Zhiyong Zhong, Yulong Liao

**Affiliations:** 1 State Key Laboratory of Electronic Thin Film and Integrated Devices, University of Electronic Science and Technology of China, Chengdu, 611731 People’s Republic of China; 2 Center for Applied Chemistry, University of Electronic Science and Technology of China, Chengdu, 611731 People’s Republic of China

**Keywords:** TiO_2_ nanotube, Crystallization, Water-assisted, Low-temperature

## Abstract

TiO_2_ nanotubes (TNTs) have drawn tremendous attention owing to their unique architectural and physical properties. Anodizing of titanium foil has proven to be the most efficient method to fabricate well-aligned TNTs, which, however, usually produces amorphous TNTs and needs further thermal annealing. Recently, a water-assisted crystallization strategy has been proposed and investigated by both science and engineering communities. This method is very efficient and energy saving, and it circumvents the drawbacks of thermal sintering approach. In this paper, we review the recent research progress in this kind of low-temperature crystallization approach. Here, various synthetic methods are summarized, and the mechanisms of the amorphous–crystalline transformation are analyzed. The fundamental properties and applications of the low-temperature products are also discussed. Furthermore, it is proved that the water-assisted crystallization approach is not only applicable to TNTs but also to crystallizing other metal oxides.
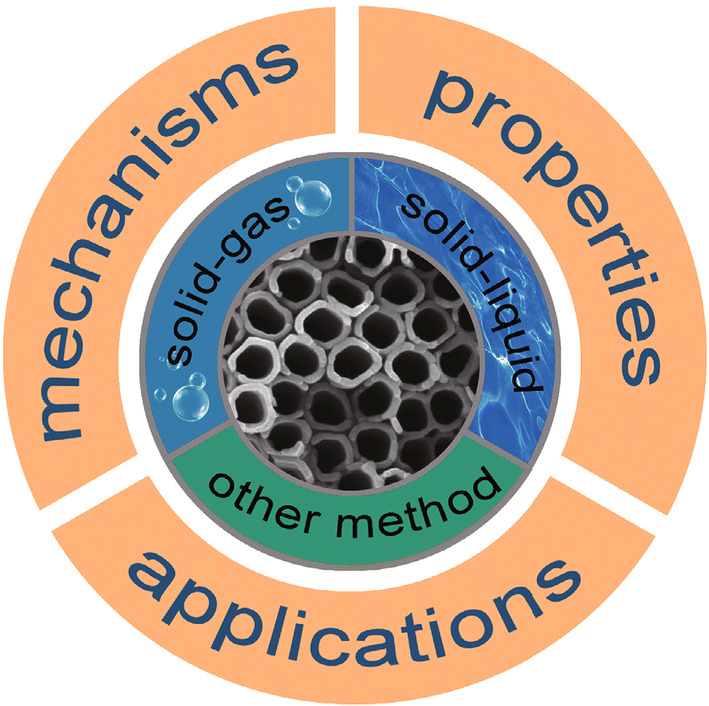

## Highlights

- This paper reviews the water-assisted crystallization for TiO_2_ nanotubes (TNTs) for the first time.
- The review summarizes various aspects of TNTs prepared by water-assisted crystallization method.

## Introduction

Over the past few decades, titanium dioxide (TiO_2_) has drawn ever-increasing interest for its application to energy and environmental areas, such as photocatalysis [1, 2], dye-sensitized solar cells (DSSCs) [3, 4], Li-ion batteries [5, 6], supercapacitors [7, 8], gas sensors, and water splitting, because of its low cost, high abundance, high chemical stability, and lack of toxicity [9–14]. In particular, one-dimensional (1-D) TiO_2_ nanotubes (TNTs) are widely investigated because they possess the advantages of both high surface area and direct carrier transport pathways, which make them a promising candidate for various fields [15–17]. Four main routes have been proposed to synthesize TNTs, including sol–gel, hydrothermal, templating, and anodic oxidation methods [18–21]. The TNTs prepared through sol–gel or hydrothermal methods are generally randomly oriented, and the templating method is limited because of its complicated fabrication process and high cost [22]. Anodic oxidation not only offers the advantages of being facile and easily scaled up but also can yield highly ordered oriented nanotube (NT) arrays, resulting in markedly enhanced performance [23–25]. In addition, such NT characteristics as the tube diameter and thickness of the tube wall can be readily manipulated by changing the experimental parameters of the anodization process [26, 27]. Therefore, anodic oxidation is expected to be a superior method for fabricating oriented TNTs.

However, the as-anodized TNTs are generally amorphous after anodization, which is not useful for many applications such as DSSCs, where the anatase phase and higher crystallinity have been proved to be essential for enhanced performance [28]. To address this issue, thermal annealing is always carried out to obtain the desired crystalline TNTs. Specifically, the conversion of amorphous TiO_2_ NTs to the anatase phase occurs at above 300 °C, and mixture of anatase and rutile appears when the annealing temperature is above 550 °C [29–31]. Although thermal annealing is an effective method to crystallize amorphous TNTs, there are some drawbacks: (1) the annealing method requires additional energy consumption and is costly; (2) this process may facilitate the formation of a thick barrier layer that separates the NT arrays from the substrate, resulting in deterioration of electron transport; (3) the high-temperature annealing process impedes the integration of NT arrays on temperature-sensitive polymeric substrates, hindering the development of lightweight TNTs-based devices [32–34]. Consequently, exploring a low-temperature method for the crystallization of TNTs is significant in broadening their applications.

In fact, there are some reports concerning the fabrication of crystallized TNTs at low temperatures without annealing [35, 36]. Su et al. found that a crystalline structure formed when the anodization was carried out at a high voltage (120 V) [37]. However, the crystallinity was so low that only a weak and broad (101) peak of anatase appeared. Ali et al. fabricated crystalline TNTs with the assistance of perchloric acid electrolytes, but the TNTs fell into the electrolyte, leading to an extra centrifugation procedure [38]. Therefore, it is urgent to find a facile strategy to crystallize the as-anodized TNTs with considerable crystallinity without involvement of any hazardous substances. In 2011, Liao et al. and Wang et al. proposed a novel water-assisted crystallization (WAC) approach to crystallize the amorphous as-anodized TNTs [39, 40]. In short, after the conventional anodization (electrolyte: ethylene glycol solution containing H_2_O and NH_4_F), the as-anodized foils were simply soaked in water for a certain time, and the amorphous TNTs arrays transformed into the anatase phase. It is truly amazing that this transformation occurred with the help of only water and without any annealing treatment or additives. Because of its facile and green chemistry features, the WAC strategy arouses people’s interest, and much effort has been devoted to it [41].

**Fig. 1:**
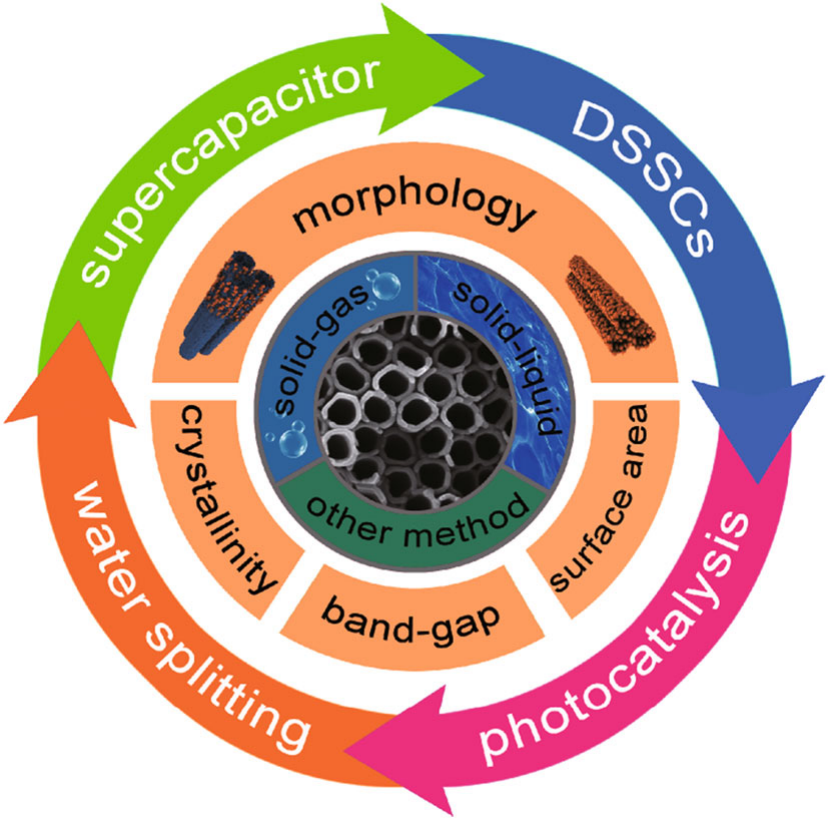
Brief description of the overall contents

Although many papers based on the WAC route have been published since 2011, no review of this field is available. Therefore, a comprehensive review could not only provide timely information for researchers but also motivate the development of TNTs. As shown in Fig. 1, this review mainly summarizes aspects of WAC for TNTs, including methods and mechanisms, fundamental properties, applications, and other materials. First, various WAC methods and the corresponding mechanisms are introduced. Second, we investigate the fundamental properties of the products after WAC treatment. Third, we introduce the main applications, including photocatalysis, DSSCs, and supercapacitors. The fourth section is mainly about other materials generated employing the WAC strategy, namely TiO_2_-based nanostructured materials and other metal oxides.

## Methods and Mechanisms

It is well known that crystallization strategy plays a key role in fabricating nanomaterials significantly influencing the structure and morphology of the products. In this section, we cover the various WAC-based crystallization methods used for synthesizing TNTs. We first introduce the water-only WAC method, in which water is the only substance employed in the crystallization process. Then, some modifications are presented, such as doping with metal ions. Finally, some ingenious methods inspired by the WAC mechanism are proposed for the preparation of TNTs. The mechanisms of the abovementioned methods are also discussed.

### Water-Only WAC Method

With respect to the water-only WAC method, the amorphous as-anodized TNTs are treated only with water without any other additives for crystallization. According to the different forms of water, it can be classified as a solid–liquid method or a solid–gas method. As the crystallization process involves only water, it is considered a green and cost-effective approach.

#### Solid–Liquid Method

**Fig. 2:**
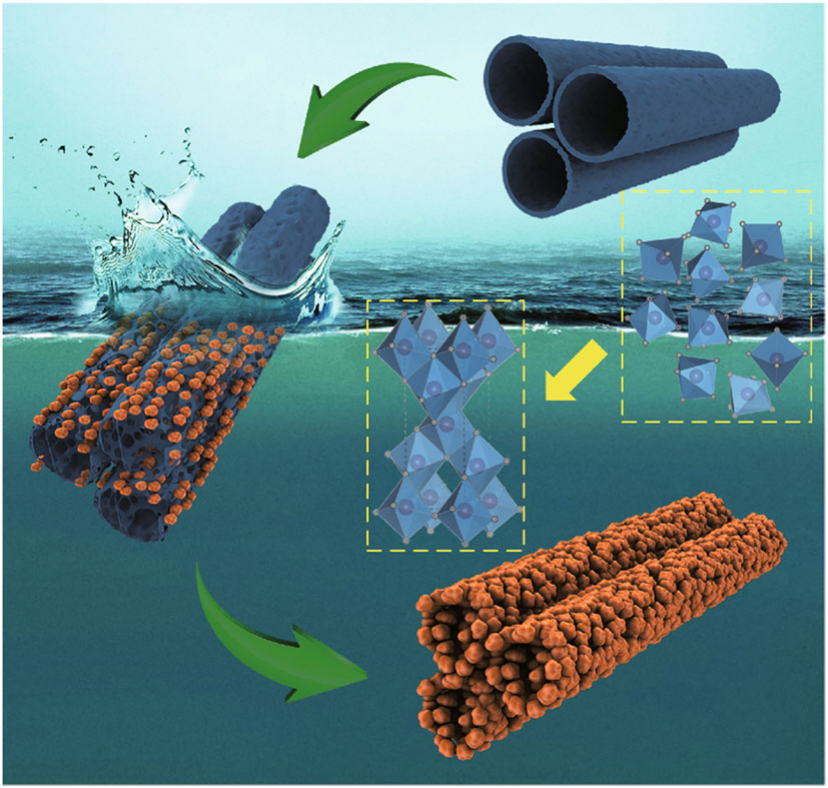
Schematic illustration of the crystallization process in water

As for the solid–liquid method, the as-anodized TNTs are simply immersed in water at different temperatures. The amorphous TNTs transform into crystalline anatase TNTs after a certain immersion time; it is schematically shown in Fig. 2. In the following sections, we mainly introduce solid–liquid methods, including room temperature (RT) water crystallization and hot water crystallization [41–49].

**Fig. 3:**
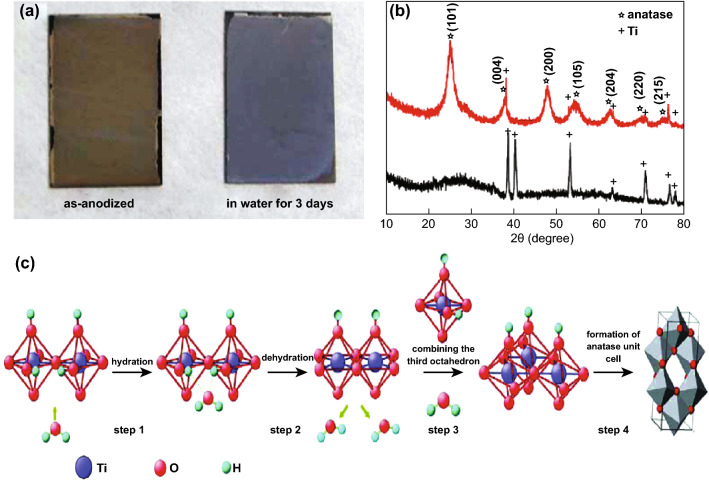
**a** Digital photographs showing the appearance change of a TiO_2_ NT/Ti film before and after the treatment. **b** XRD patterns demonstrating the phase transformation before (black line) and after (red line) the water soaking for the TiO_2_ NT/Ti film. **c** Schematic showing the nucleation of anatase from amorphous TiO_2_ induced by water [40]. Copyright © 2011 American Chemical Society. (Color figure online)

In 2011, Wang et al. prepared amorphous TNTs by anodization (electrolyte: ethylene glycol solution containing NH_4_F and H_2_O) and then immersed them in water at RT (~ 25 °C) [40]. As a result, the amorphous TNTs transformed into pure anatase phase after 3 days without occurrence of rutile or brookite phases (Fig. 3a, b). In addition, the obtained anatase TNTs were stable, and no discernible changes were observed when the immersion time was prolonged to 30 days. The amorphous TNTs transformed into the anatase phase not only by annealing but also through the water-assisted strategy at RT. Based on systematic studies, a dissolution–precipitation mechanism was proposed to explain this type of WAC phenomenon. The building blocks of the amorphous materials are randomly distributed, while the crystallized materials are long-range ordered [50]. As TiO_6_ octahedra are the building blocks for both amorphous TNTs and anatase TNTs, it is assumed that the amorphous–anatase transformation is a process that rearranges the TiO_6_ octahedra with the assistance of water. As schematically shown in Fig. 3c, two different TiO_6_ octahedra, which share one common vertex, first absorb a water molecule forming a bridge between the surface hydroxyl groups through the lone electrons on the oxygen (step 1). In step 2, dehydration of the abovementioned complex occurs. Two water molecules are ejected and one oxygen atom is taken away by forming a new water molecule, resulting in the linkage of octahedra by sharing one common edge. Subsequently, third octahedra proceeds in a similar hydration-dehydration process and the three octahedra become linked together by sharing their edges at a right angle (step 3). Finally, the right-angle assembly connects with another identical assembly, leading to the basic unit cell of anatase TiO_2_ (step 4). The reason why the rearrangement of TiO_6_ octahedra leads to the formation of the anatase phase and not the rutile phase can be explained as follows. It is believed that the Gibbs free energy of anatase clusters is lower than that of rutile clusters; therefore, the anatase phase is more thermodynamically stable [51]. It is concluded that the amorphous–anatase transformation is achieved through a dissolution–precipitation process, in which randomly distributed TiO_6_ octahedra are rearranged with the assistance of water. Owing to the dissolution and reprecipitation processes, the morphology of TNTs cannot stay the same as before, and numerous pores are formed, which will be discussed in the next chapter.

**Fig. 4:**
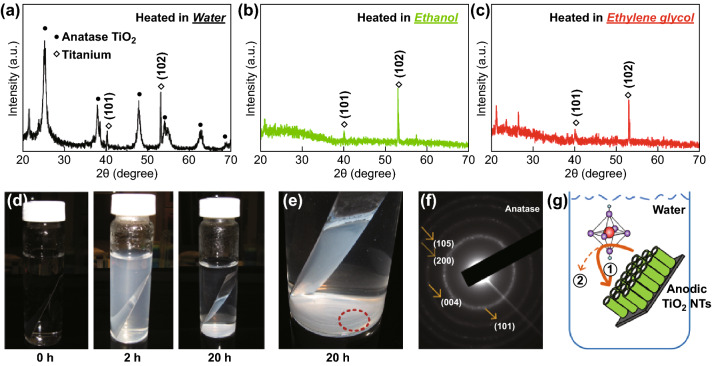
XRD patterns of anodic TiO_2_ NT array films crystallized in **a** water, **b** ethanol, and **c** ethylene glycol at 90 °C for 20 h. **d** Digital photographs of the treatment system at 90 °C with treatment duration rising from 0 to 20 h. **e** Magnified digital photograph of the 20-h sample. **f** Selected area diffraction pattern of the precipitated TiO_2_ nanoparticles, indicating the anatase phase of the precipitated nanoparticles [52]. **g** Schematic diagram of the dissolution-recrystallization-precipitation mechanism. Copyright © 2016 American Chemical Society

Although the dissolution–precipitation process has been developed, there are still many scientific issues to be explored. In 2016, we further investigated the amorphous–anatase transformation and supplemented the dissolution–precipitation mechanism by immersing the as-anodized TNTs in hot water (90 °C) for a certain time [52]. We named it the WAC strategy because water is the only substance involved in this crystallization process. Could any other substance also lead to this amazing amorphous–anatase transformation under the same conditions? In addition to immersing the amorphous TNTs in hot water, we also immersed them in ethanol and ethylene glycol solutions. However, no distinct anatase peaks were detected except for the Ti substrate peaks, as shown in Fig. 4b, c. We inferred that water is an essential factor in the crystallization process, confirming the water-assisted mechanism. In addition, we not only focused on the TNTs but also paid attention to the byproducts leading to deeper insight into the WAC mechanism. Specifically, after the samples were immersed for 2 h at 90 °C, the water became turbid owing to the existence of white precipitate byproducts. When the immersion time was prolonged to 20 h, white products settled to the bottom, and the water became transparent again, as shown in Fig. 4d, e. The selected area electron diffraction (SAED) results in Fig. 4f reveal that the white byproducts are composed of nanoparticles (NPs) possessing an anatase crystalline structure in line with that of the TNTs on Ti substrate. Based on this result, a supplementary dissolution–recrystallization-precipitation mechanism was proposed; its schematic diagram is presented in Fig. 4g. First, TiO_6_ octahedra dissolve in water forming Ti(OH)_6_^2−^ species. Then, the Ti(OH)_6_^2−^ species spontaneously recrystallize and precipitate in situ into TNTs, maintaining the mechanical nanotubular structure. However, a fraction of Ti(OH)_6_^2−^ species recrystallize and precipitate into anatase TiO_2_ NPs, which are apart from the TNTs and suspended in water. In other words, the overall reactions of the amorphous–anatase transformation can be described as follows:

1 $${\text{TiO}}_{{2({\text{am}})}} + 4{\text{H}}_{2} {\text{O}} \to {\text{Ti}}\left( {\text{OH}} \right)_{6}^{2 - } + 2{\text{H}}^{ + }$$

2 $${\text{Ti}}\left( {\text{OH}} \right)_{6}^{2 - } + 2{\text{H}}^{ + } \to {\text{TiO}}_{{2({\text{an}})}} + 4{\text{H}}_{2} {\text{O}}$$

Here, TiO_2(am)_ represents the amorphous TNTs, and TiO_2(an)_ represents the anatase TNTs. These findings corroborate and enrich the water-assisted dissolution–precipitation mechanism and provide deeper insight into the WAC strategy.

It is worth noting that experimental parameters, such as water temperature and immersion time, have a significant influence on the properties of the obtained anatase TNTs. For example, Karine et al. employed the solid–liquid method to crystallize amorphous TNTs for various time periods at different temperatures (RT, 80, 100, and 120 °C). They found that the crystallographic properties and morphological features are distinctive under different experimental conditions [46]. Actually, the solid–liquid method to crystallize amorphous TNTs is widely recognized as a fundamental and convenient strategy. The dissolution–precipitation mechanism is not only applicable to the solid–liquid method but also serves as the theoretical basis of other derived synthetic strategies.

#### Solid–Gas Method

Although the solid–liquid WAC method is cost-effective and convenient, the full-of-water condition results in a disadvantage: TNT films may detach because of the dissolution of the bottom layer under the NTs. To circumvent this problem, a solid–gas method, in which water is gaseous, is proposed [44, 53, 54].

**Fig. 5:**
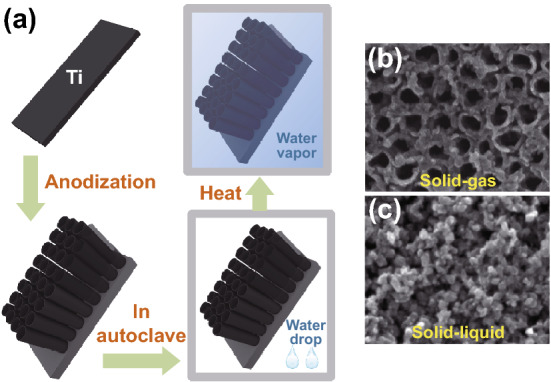
**a** Flowchart for the low-temperature crystallization of amorphous TiO_2_ nanotubular arrays by solid–gas reaction. In a Teflon-lined stainless autoclave, the as-anodized amorphous TiO_2_ nanotubes fabricated by anodization reacted with water vapor to yield anatase phase. Morphology images of TiO_2_ nanotubular arrays prepared by anodizing Ti foil at 20 V for 20 min in 0.5 wt% HF solution, followed by **b** hydrothermal solid–gas crystallization and **c** hydrothermal solid–liquid crystallization at 160 °C for 4 h [54]. Copyright © 2013 The Royal Society of Chemistry

As schematically shown in Fig. 5a, the amorphous TNTs are put into a Teflon-lined stainless autoclave containing a small amount of water. With increasing temperature, the liquid water turns into water vapor surrounding the TNTs and crystallizes them. Liu et al. employed the solid–gas method to crystallize the amorphous TNTs at temperatures of 130–180 °C, and only 0.3 mL water was added to the autoclave [54]. The dissolution–precipitation process proceeded at the TNT/vapor interfaces leading to the crystallization of amorphous TNTs. X-ray diffraction (XRD) results showed this amorphous-anatase transformation, and the effects of vapor temperature and crystallization duration were also investigated. For comparison, they also used the solid–liquid method to crystallize amorphous TNTs by immersing them in water (maintaining other conditions the same). At temperatures of 130 or 160 °C, the nanotubular structure of the solid–gas samples was preserved, and only some NPs appeared on tube walls (Fig. 5b), which is a common morphology feature resulting from the WAC method. In contrast, the solid–liquid samples exhibited a serious collapse of TNTs, and the nanotubular architecture was destroyed (Fig. 5c), which is consistent with previous reports. These results indicate that the solid–gas method can alleviate the collapse of NTs, possibly because of relatively slow TNT/vapor interface reactions.

To compare with the abovementioned example, a facile solid–gas method at a low temperature of 50 °C was proposed by Andrea et al. [55]. In this case, the temperature of the TNT arrays was not the same as that of the water vapor because they were not sealed in an enclosed space. Specifically, the as-anodized samples were fixed into a clamping system and exposed to water vapor that had been previously heated to 90 °C. No intentional heating was provided to the TNTs, and their temperature reached 50 °C (measured by a Pt100 temperature detector) resulting from contact with the heated vapor. The researchers demonstrated that a thin water layer emerges in this situation and subsequently forms part of a solid/liquid/vapor interface system where the water-assisted dissolution–precipitation occurred. After exposure for only 20 min, (101) peaks belonging to the TiO_2_ anatase phase were detected in the XRD results, in contrast with the prior amorphous nature. This result shows that amorphous TiO_2_ can transform into the anatase phase even at a low temperature of 50 °C. Considering the presence of water vapor in air at RT, we may wonder if the amorphous–anatase transformation would occur when the amorphous TiO_2_ is exposed to air. Su et al. prepared amorphous TiO_2_ and placed it in air to observe the changes [56]. As a result, the amorphous TiO_2_ transformed into the anatase phase within 90 days. They attributed this phenomenon to the rearrangement of TiO_6_ octahedra with assistance from the water from moisture. Although the crystallization period was extremely long, this result still indicates that the amorphous–anatase transformation occurs in air at RT and normal atmosphere pressure without any solvent or additive.

The water-only WAC method can effectively crystallize amorphous TNTs. The dissolution–precipitation process plays an important role in this amorphous–anatase transformation in which TiO_6_ octahedra are rearranged with the assistance of water.

### Modified WAC Method

In addition to the water-only WAC method, much effort has been devoted to investigating other modified methods that employ aqueous solutions containing various ions. In this section, we introduce the modified WAC methods involving aqueous solutions containing metal ions and nonmetal ions [57–60].

#### Aqueous Solution Containing Metal Ions

Since the first report of TNTs, many efforts have been devoted to doping TNTs with metal ions [61–66]. Although the doped TNTs exhibit satisfying performances, the complicated procedures of generating them limit their development. Inspired by the WAC strategy, researchers used aqueous solutions containing metal ions to crystallize the amorphous TNTs and dope the metal elements into TiO_2_ at the same time. For example, Zhang et al. fabricated MTiO_3_ (M = Zn, Co, Ni) NTs by a hydrothermal treatment that immersed the amorphous TNTs into aqueous solutions containing different metal acetates [67]. Considering the participation of metal acetates, the reactions in the autoclave can be described as follows:

3 $${\text{M}}\left( {\text{Ac}} \right)_{2} \to {\text{M}}^{2 + } + 2{\text{Ac}}^{ - }$$

4 $${\text{Ac}}^{ - } + {\text{H}}^{ + } \to {\text{HAc}}$$

5 $${\text{Ti}}\left( {\text{OH}} \right)_{6}^{2 - } + {\text{M}}^{2 + } \to {\text{MTiO}}_{3} + 3{\text{H}}_{2} {\text{O}}$$

**Fig. 6:**
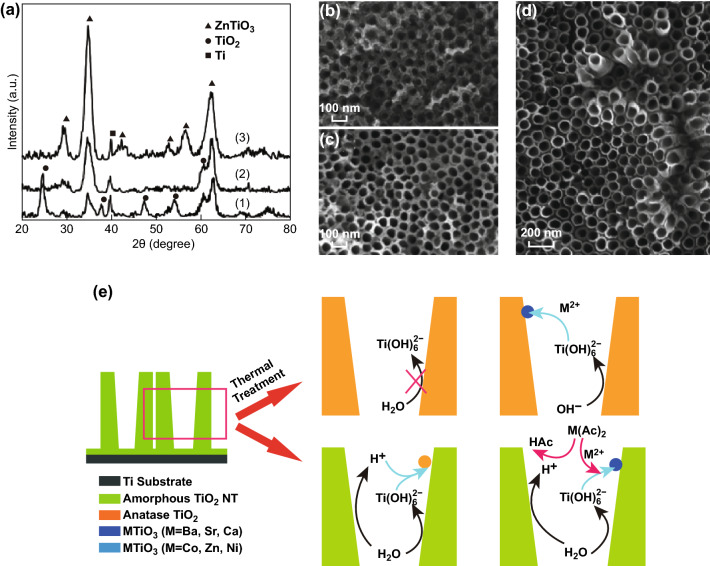
**a** XRD patterns of the as-hydrothermal samples after annealing at 450 °C for 3 h in air (lines 1, 2, 3 represent the 0.05 M, 0.2 M, and 0.5 M Zn (Ac)_2_ solutions). SEM images of the samples hydrothermally treated with Zn(Ac)_2_ solutions with different concentrations at 200 °C for 6 h: **b** 0.05 M and **c** 0.5 M. **d** SEM image of anatase TNTs after hydrothermal treatment in 0.2 M Zn (Ac)_2_ at 200 °C for 6 h [67]. **e** Schematic illustration of the reaction processes in the presence of anatase or amorphous TNTs. Copyright © 2014 The Royal Society of Chemistry

Here, M represents the metal ions, and Ac represents acetate (CH_3_COO^−^). As mentioned above, the amorphous–anatase transformation is highly dependent on the reaction between Ti(OH)_6_^2−^ and H^+^ (Eq. 2). From Eqs. 3 and 4, however, we can see that H^+^ is consumed because of the presence of Ac^−^. Consequently, the reaction in Eq. 2 is hindered, and the combining of Ti(OH)_6_^2−^ and M^2+^ occurs and is promoted. As shown in Fig. 6a, the XRD results confirm the existence of ZnTiO_3_ when the metal acetate is Zn(Ac)_2_. Actually, the employment of an aqueous solution of Zn(Ac)_2_ not only causes the formation of ZnTiO_3_ but also influences the morphology of NTs. Some NPs emerge during the dissolution–precipitation process when the water-only WAC method is used because of the reaction in Eq. 2. These NPs are usually adhered on tube walls and form a NP/TNT morphological feature. In Zhang’s work, the NP/TNT structure appeared when the concentration of Zn(Ac)_2_ was low (0.05 M), as shown in Fig. 6b. In contrast, the nanotubular architecture remained intact, and few NPs were observed when the concentration was increased to 0.5 M (Fig. 6c). These results confirm that the addition of M(Ac)_2_ hinders the combining of Ti(OH)_6_^2−^ and H^+^ and, subsequently, alleviates the collapse of NTs. To further investigate the reactions and obtain deep insights, additional experiments were carried out. On the one hand, crystalline anatase TNTs, instead of the amorphous TNTs, were hydrothermally treated with 0.2 M Zn(Ac)_2_. As expected, ZnTiO_3_ was not detected in this case. Because the stability of anatase TiO_2_ is relatively high, the TiO_6_ octahedra do not absorb water molecules and form Ti(OH)_6_^2−^ groups [68–70]. Therefore, the reactions in Eq. 5 are restricted, and ZnTiO_3_ is not obtained. On the other hand, ZnTiO_3_ was also not obtained when Zn(Ac)_2_ was replaced by ZnCl_2_ and the other conditions were maintained, which could be attributed to the fact that strong acid radicals would not proceed the reactions, as in Eq. 4. This result demonstrates that both the amorphous nature and weak acid radicals are essential in the preparation of MTiO_3_. In addition, the morphology of the anatase TNTs was nearly unchanged after hydrothermal treatment in 0.2 M Zn(Ac)_2_ solution (Fig. 6d). Based on the above results, a schematic illustration of the reactions in the presence of anatase or amorphous TNTs is displayed in Fig. 6e.

In contrast to the metal elements used for doping, some metal ions in the solution nucleate and crystallize simultaneously with the recrystallization of amorphous TNTs. For instance, Zhao et al. fabricated Ag-TiO_2_ nanocomposites by immersing amorphous TNTs in an aqueous solution containing AgNO_3_, glucose, and ethanol at 180 °C [71]. After 2 h, the sample contained three phases, including silver, anatase TiO_2_, and titanium (substrate). In this reaction, Ag^+^ ions diffused into the amorphous TNTs and transformed into Ag NPs. The WAC of amorphous TNTs proceeded at the same time, eventually resulting in Ag-TNT nanocomposites.

#### Aqueous Solutions Containing Nonmetal Ions

**Fig. 7:**
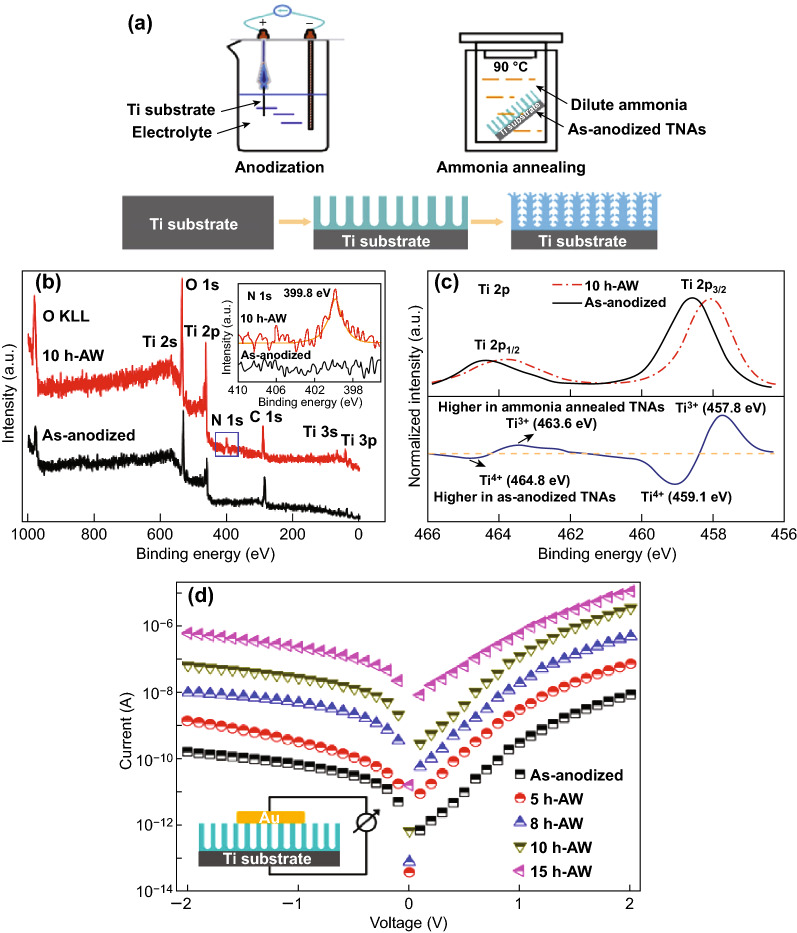
**a** Schematic diagram showing the fabrication of crystalline TNTs by immersing the as-anodized sample in the solution of ammonia. **b** Low-resolution XPS spectra of as-anodized TNAs and ammonia-treated TNAs (10 h). The inset shows the corresponding N 1s core-level XPS spectra with high resolution. **c** The corresponding normalized Ti 2p core-level XPS spectra with high resolution together with their difference spectrum (‘ammonia-treated TNAs’ minus ‘as-anodized TNAs’). **d** *I*–*V* characteristics of the as-anodized TNAs and ammonia-treated TNTs annealed for 5, 8, 10, and 15 h, respectively. The inset: a simplified sketch of the two-point measurement arrangement [78]. Copyright © 2014 Elsevier B.V

As for TNTs with nonmetal ions, we mainly discuss nitrogen-doped TNTs (N-TNTs), which have many advantages in various fields [72–75]. A variety of strategies, such as ammonolysis and ion implantation methods, have been developed to implant nitrogen into TNTs [76, 77]. Unfortunately, high temperature is usually an inevitable experimental condition for obtaining N-TNTs limiting their development for many applications. Consequently, it is important to develop a convenient low-temperature method to prepare N-TNTs. Wang et al. fabricated N-TNTs by immersing the as-anodized amorphous TNTs into an aqueous solution of ammonia at 90 °C, as shown in Fig. 7a [78]. Because of the weak alkaline environment of the solution, the reactions in Eqs. 1 and 2 were accelerated. As expected, the amorphous TNTs transformed into the anatase phase after immersion, and the crystallinity increased with increasing immersion time. As shown in Fig. 7b, X-ray photoelectron spectroscopy (XPS) was carried out to investigate the influence of ammonia. Sharp peaks for Ti, O, and C were detected in both the as-anodized and ammonia-treated samples, while the N 1s peak was also observed in the latter. The N 1s peak located at approximately 399.8 eV was assigned to interstitial nitrogen with a Ti–O–N structure, which is consistent with other studies [79, 80]. The normalized Ti 2p core-level XPS spectra of the as-anodized and ammonia-treated samples are presented in Fig. 7c. The peak of the ammonia-treated sample is clearly shifted compared with that of the as-anodized sample, indicating that their Ti ions have different bonding environments. The researchers demonstrated that this redshift could be attributed to an increase in electron cloud density on Ti owing to the presence of nitrogen. The XPS results also confirm that there were more Ti^3+^ ions in the ammonia-treated sample, and we can conclude that oxygen vacancies (Ti^3+^) emerge during the ammonia solution immersion. Moreover, the N-TNTs exhibit an elevated conductivity, as shown in Fig. 7d. The conductivity of the as-anodized TNTs was only 8.96 × 10^−9^ S m^−1^, while this value of the ammonia-treated sample reached as high as 7.42 × 10^−6^ S m^−1^. The researchers demonstrated that the variation in conductivity could be attributed to the enhanced crystallinity and oxygen vacancies introduced in the ammonia-treated products.

In addition, Cui et al. immersed the as-anodized TNTs into an aqueous solution of various concentrations of (NH_4_)_2_TiF_6_ (0.005, 0.01 and 0.02 M) [81]. Although water molecules were considered to be the main agents in the crystallization of TNTs because of the very low concentrations of (NH_4_)_2_TiF_6_, the presence of [NH_4_]^+^ and [TiF_6_]^2−^ markedly influenced the products, especially their morphology. When these materials were employed in supercapacitors, the specific capacitance of the (NH_4_)_2_TiF_6_-treated sample was three times that of the sample without (NH_4_)_2_TiF_6_ treatment.

### Other Methods

**Fig. 8:**
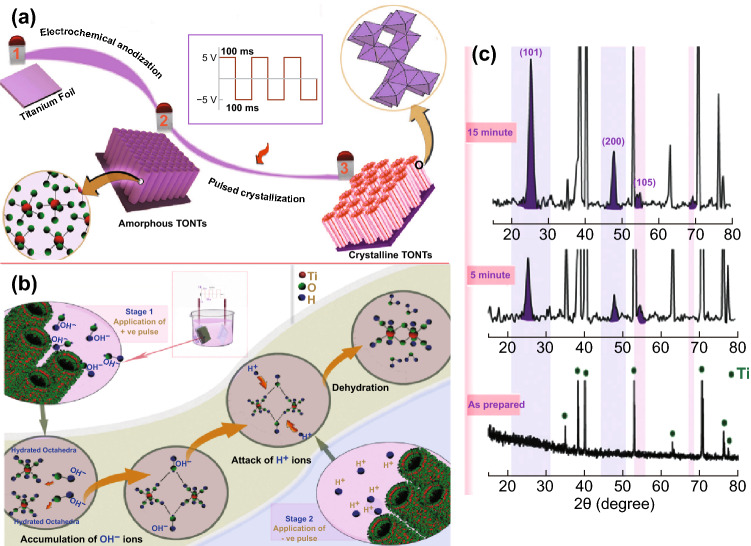
**a** Schematic illustration of the crystallization process at room temperature: first step: electrochemical anodization; second step: pulsed crystallization. **b** The mechanism of the superfast room temperature crystallization of TNTs. **c** XRD patterns of the as-anodized and pulsed-crystallized TNTs [82]. Copyright © 2017 The Royal Society of Chemistry

We have introduced the main WAC methods, and a dissolution–precipitation mechanism has been adopted to explain the amorphous-anatase transformation. However, this transformation process usually requires a relatively long crystallization time, especially for the solid–liquid method. At RT, days are needed to achieve the transformation because of the low dissolution–precipitation rate. Therefore, accelerating the WAC process becomes a challenging issue. In 2017, Aijo et al. reported a very efficient technique for RT crystallization of as-anodized TNTs, which is quite different from the methods above [82]. The most attractive feature of this method is the very fast amorphous-anatase transformation, which only requires 5 min. As shown in Fig. 8a, the preparation process comprises the following steps: (1) titanium foil is anodized in an ethylene glycol solution containing ammonium fluoride and water; (2) the sample is crystallized using a two-electrode system, where the as-anodized sample is used as the working electrode and platinum acts as the counter electrode. An alternating square voltage pulse with a pulse width of 100 ms is employed, and the electrolyte used in this step is a 1 M KCl aqueous solution. The addition of KCl improves the conductivity, and this species does not participate in the reactions on either the anode or the cathode. After a pulse treatment of only 5 min, a sharp (101) XRD peak of the anatase phase was observed, suggesting successful amorphous–anatase transformation in a short time. In addition, the nanotubular structure was well maintained after pulse treatment, and few NPs were detected, in contrast with the water immersion samples, in which many NPs adhered to tube walls. As shown in Fig. 8b, an electrophilic-nucleophilic mechanism is proposed to explain the crystallization process. In the first stage, a positive pulse (+ 5 V) is applied to the amorphous TNT electrode, making it electrophilic and leading to the accumulation of OH^−^ ions on the surface. These OH^−^ ions act as ‘bridges’ bonding the two adjacent Ti(OH)_6_^2−^ octahedra together. In the second stage, a negative pulse (− 5 V) is applied to the amorphous TNT electrode, making it nucleophilic and causing the attraction of H^+^ ions. These H^+^ ions ‘attack’ the ‘bridges,’ resulting in the formation of edge-shared octahedra. With the increase in pulse treatment time, this process continues until a basic unit of anatase TiO_2_ is formed.

In summary, there are many ways to accomplish the WAC of amorphous TNTs. The dissolution–precipitation mechanism is appropriate for most of the methods; some other mechanisms were also proposed. Finding a green, efficient, and low-cost method for crystallizing amorphous TNTs not only promote the further investigation of TiO_2_ but also have great significance for the exploration of crystallography.

## Fundamental Properties

After the WAC process, some properties of the products are quite different from those of the as-anodized and high-temperature-annealed samples. In this section, we mainly introduce fundamental properties, including the morphology, surface area, crystallinity, and bandgap, of the products prepared by WAC methods.

### Morphology Evolution

It is well known that the morphology of nanomaterials is the most important factor affecting their properties. Although the 1-D structure usually does not collapse completely during the WAC, there are still many changes that cannot be ignored. In the following, these new morphological features and the corresponding influence factors are summarized.

#### NT-NP/NT-NR Evolution

**Fig. 9:**
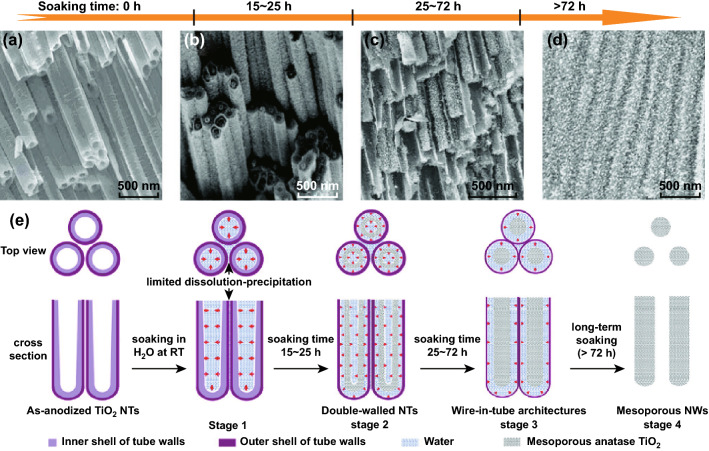
SEM characterization of the morphological evolution as a function of water soaking time: **a** as-anodized TiO_2_ nanotube arrays, **b** intermediate double-walled TiO_2_ nanotube arrays obtained by water soaking for 15–25 h, **c** intermediate wire-in-tube TiO_2_ nanoarchitecture formed by water soaking for 25–72 h, and **d** mesoporous TiO_2_ nanowire arrays obtained by a long-term water soaking (> 72 h). **e** Scheme illustrating the morphology transformation of the as-anodized TiO_2_ nanotubes upon water soaking. The red arrows denote the direction along which the dissolution–precipitation of TiO_6_ octahedra proceeds [40]. Copyright © 2011 American Chemical Society. (Color figure online)

*Treatment time* Figure 9a–d shows the scanning electron microscopy (SEM) images of the morphological evolution of a sample as a function of water immersion time. From Fig. 9a, it can be seen that the as-anodized TNTs have smooth tube walls. However, when the sample is immersed in water at RT for 30 h, some NPs with a mean diameter of approximately 10 nm appear on the tube walls forming an NP/NT structure. With increasing immersion time, more NPs emerge on both the inner side and outer side of the tube walls. It should be noted that the NP/NT structure is still maintained, because the inside of the tube is not completely filled with NPs. If we further prolong the immersion time to over 72 h, the original tubular structure is no longer visible, and only the solid nanorods (NRs) are detected (Fig. 9d). The NT-NP/NT-NR evolution can be attributed to the dissolution–precipitation, in which TiO_6_ octahedra from the original NTs dissolve, rearrange, and precipitate as anatase TiO_2_ NPs on tube walls. In Fig. 9e, a scheme illustrating the transformation from amorphous NTs to anatase NRs is presented. In addition, Wang et al. found that a double-walled NT structure forms before the formation of NPs/NTs (Fig. 9b) [40]. It is believed that the large space inside the NTs facilitates water molecules to access the inner surfaces of the tubes, while the narrow space between the adjacent NTs limits the transport of water molecules. Therefore, the dissolution–precipitation process occurring on the outer surfaces of the NTs is dramatically slow compared with that on the inner side, finally forming the double-walled structure.

**Fig. 10:**
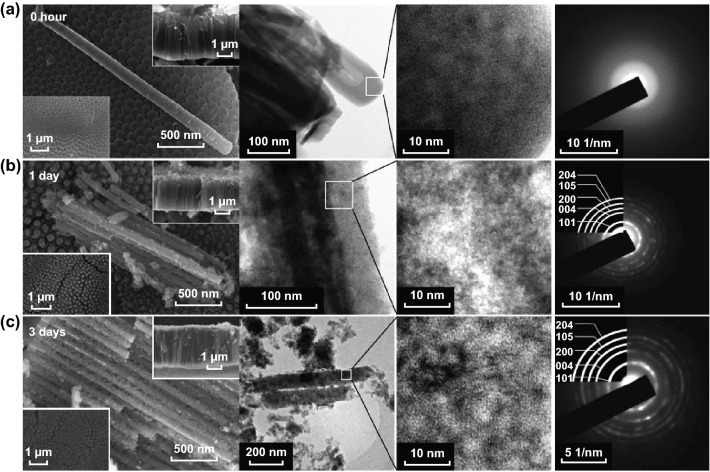
SEM, HRTEM and SAED images of the **a** as-anodized TNTs, **b** after water soaking for 1 day, and **c** after water soaking for 3 days [83]

This type of NT-NP/NT-NR evolution has also been discovered in most experiments using WAC methods. For example, Cao et al. immersed the as-anodized TNTs into water for different duration times (up to 7 days) and investigated the morphology transformation [83]. Figure 10 shows SEM and transmission electron microscopy (TEM) images of the samples with different immersion times. For the as-anodized TNTs (Fig. 10a), the tube wall is smooth, and no lattice fringes or diffraction rings can be detected from the high-resolution TEM and SAED images, confirming the amorphous nature. After immersion for 24 h (Fig. 10b), an NP/NT architecture appears, and the diffraction rings assigned to anatase TiO_2_ are observed. Within 3 days (Fig. 10c), the NPs/NTs transforms into NRs composed of NPs. Both the lattice fringes and diffraction rings are clearly observed, indicating the good crystallinity of the prepared anatase TNTs. It can be noted that a higher surface area can always be obtained after WAC treatment, resulting from the formation of pores and nanoparticles during the morphology evolution process. On the other hand, losing the tubular structure also causes some drawbacks listed as follows: (1) numerous boundaries between nanoparticles lead to a slow diffusion of electrons; (2) the structure may be not as robust as before. In short, there are advantages and disadvantages of the morphology evolution, and it can be controlled to meet different requirements.

*Treatment temperature* Regarding the solid–liquid method, its high temperature (> 100 °C) can easily lead to the collapse of TNT films [53]. In contrast, the TNT films are relatively robust under solid–gas treatment. Additionally, the treatment time plays a key role in the NT-NP/NT-NR transformation. Liu et al. demonstrated that high temperature accelerates the dissolution–precipitation rate and, subsequently, facilitates the formation of NP/NT structures [54]. After exposure of the sample to water vapor for 4 h at 130 °C, only a small number of NPs appeared, and the nanotubular structure was well preserved. Nevertheless, the nanotubular structure of the 180 °C sample started collapsing, and the NR structure was about to form.

*NT lengths* Although many researchers have studied the influences of treatment time and temperature, little attention has been paid to the effect of NT length. In 2013, Wang et al. used the solid–liquid WAC method to crystallize the amorphous TNTs and investigated the influence of NT lengths on the morphology [84]. When as-anodized TNTs with lengths of 3.5 µm were immersed in water for 45 min, only a few NPs were observed on surfaces, and the tube walls were still smooth. For the 6.5 µm samples, many more NPs were generated, and the tube walls became extremely rough forming a typical NP/NT structure. For the longest tubes (16.5 µm), solid NRs composed of NPs appeared. This result shows that the long NTs are more prone to NT-NP/NT-NR evolution than the short NTs, which may be attributed to the abundant titania source allowing the dissolution–precipitation process to occur. Furthermore, it was also found that the morphology near the tube bottom was different from that of the tube top. Specifically, the bottom NTs were filled with NPs and became NRs, while the top NTs still exhibited an NP/NT structure. The solid NR structure of the bottom part of NTs limits the attachment and transportation of dye molecules, which is the reason why the amount of dye loading does not increase dramatically with longer NTs.

*pH effect* From Eq. 2, we can see that the concentration of H^+^ ions plays a key role in the dissolution–precipitation and, subsequently, affects the formation of TiO_2_ NPs. Zhang et al. investigated the influence of pH on morphology transformation and found that the NP size is strongly related to the pH value [67]. In HCl solution (pH = 3), the NTs transformed into NRs composed of compact NPs with a diameter of approximately 80 nm. In contrast, the NRs formed in deionized water (pH = 6.5) were composed of NPs with a diameter of approximately 40 nm. When the pH was 11 (NaOH solution), smaller NPs of 20–30 nm in diameter were observed on both the top surface and tube walls. It can be concluded that the NP size decreases with increasing pH value; in other words, the NPs grow larger with a higher concentration of H^+^. This phenomenon can be explained by the fact that the presence of more H^+^ ions at low pH accelerates the reaction in Eq. 2, thus, forming larger anatase TiO_2_ NPs. This theory also applies to the condition when the amorphous TNTs are immersed in a solution containing weak acid radicals such as acetate (CH_3_COO^−^). CH_3_COO^−^ can combine with H^+^ to restrict the reaction in Eq. 2; therefore, the formation of NPs is hindered, and the nanotubular structure is usually maintained.

Besides, there are many other factors influencing the NT-NP/NT-NR morphology evolution. For example, Huo et al. reported that the dissolved oxygen in water remarkably affects the tubular structure of TNTs. When the dissolved oxygen was eliminated by purging with N_2_, and then the WAC procedure was conducted while maintaining other conditions the same, the nanotubular architecture was well kept and the NT-NP/NT-NR evolution did not occur [43]. In addition, the residual fluorine on TNTs after anodization also affects the morphology transformation. The existence of fluorine accelerates the NT-NP/NT-NR evolution because the titanium fluoride compounds can create anatase TiO_2_ by hydrolysis [83, 85, 86] In summary, NP/NT and NR structures are commonly observed because of the dissolution–precipitation process during crystallization. There is no doubt that the participation of NPs would greatly increase the surface areas of products, which is beneficial for many applications.

#### Other Morphologies

**Fig. 11:**
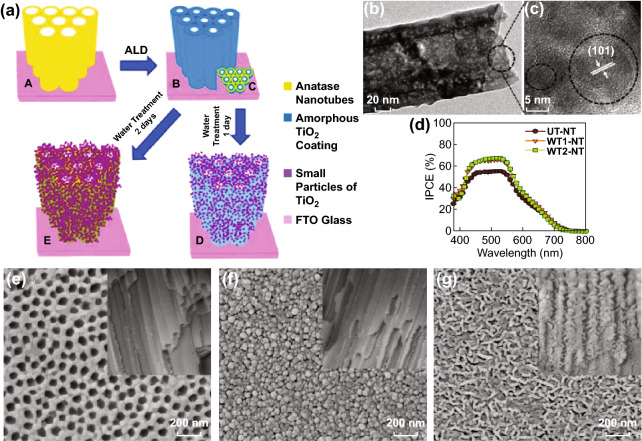
**a** Schematic illustration of the fabrication stages of the nanoparticle decorated TiO_2_ NT array photoanode: (A) untreated TiO_2_ NT array on FTO substrate (UT-NT), (B) ALD amorphous TiO_2_ thin layer coated on the TiO_2_ NT array (AT-NT), (C) cross-sectional view of (B), (D) AT-NT electrode post-water treated for 1 day (WT1-NT), and (E) AT-NT electrode water treated for 2 days (WT2-NT). **b** TEM image of the sample after water treatment. **c** The corresponding HRTEM lattice images of the circled portions in **b**. **d** Incident photoconversion efficiency spectra of DSSCs using untreated and water-treated TiO_2_ NT array photoanodes. SEM images of **e** as-anodized TNTs, **f** after being immersed in water at 90 °C for 8 h, and **g** after being immersed in ammonia solution (the concentration of Vol_ammonia_/Vol_water_ = 1:1) at 90 °C for 8 h [45, 75]. Copyright © 2013 The Royal Society of Chemistry and 2014 Elsevier Masson SAS

*Advanced NPs/NTs* As the crystallization using the WAC method is always accompanied with the formation of particles, it is considered as a convenient way to decorate NPs on NTs, in contrast with the presynthesized NP method [87–90]. However, the generation of NPs is due to the sacrifice of NTs, which makes the tube walls become gradually thinner during the treatment. With this sacrifice, the nanotubular structure may completely collapse and transform into NRs. Therefore, it is a challenge to decorate NPs on tube walls without the destruction of NTs. Kurian et al. proposed a strategy of coating a secondary thin amorphous TiO_2_ layer on previously crystallized TNTs and then immersing them in water [45]. As illustrated in Fig. 11a, crystallized TNTs were first obtained by anodization and annealing and then transferred onto the fluorine-doped tin oxide (FTO) substrate. Second, a thin amorphous TiO_2_ layer (approximately 15 nm in thickness) was deposited on the TNT film by the atomic layer deposition (ALD) technique. Finally, the resultant sample was immersed in water for different durations. The primary crystallized anatase TNTs are quite stable when immersed in water [91]. Therefore, when the ALD-treated sample was immersed in water, the outer amorphous layer transformed into anatase TiO_2_ NPs through the dissolution–precipitation process, while the inner crystallized NTs remained unchanged. As shown in Fig. 11b, c, some crystalline NPs are decorated on the tube walls. Because the NTs are not destroyed at all, we define this type of NP-decorated structure as advanced NPs/NTs. The prepared products were employed in DSSCs, and they showed a better performance than the sample without water treatment (Fig. 11d). There are two reasons that explain this promotion: (1) the participation of NPs dramatically increases the surface area and is beneficial for higher dye loading; and (2) the primary crystallized NTs serve as backbones for electron transport. It is believed that this strategy can be applied to various 3-D structures to both increase the surface area and promote the dye loading amount without destroying the geometric architecture.

*Nanoworms/NTs* The water-only method usually causes NP/NT and NR structures to form, as mentioned above. Some novel morphological features appear when other ions are involved in the solution. For example, a nanoworm/NT structure was obtained when the as-anodized TNTs were immersed in the aqueous solution of ammonia at 90 °C [75]. Compared with the normal nanotubular structure of the as-anodized sample (Fig. 11e), many NPs appear on the top surface of the sample immersed in hot water (Fig. 11f), in line with previous reports. However, when the as-anodized TNTs were immersed in an aqueous solution of ammonia, many worm-like titania structures with a length of approximately 100 nm form on both the top surfaces and tube walls of TNTs, as shown in Fig. 11g. From the cross-sectional SEM image, it is clear that the diameter of the NTs decreases substantially after immersion. When the concentration of ammonia increased, the worm-like morphological feature was maintained and became denser. Researchers found that this type of nanoworm/NT structure exhibits a better performance for degrading methyl orange (MO) than the high-temperature-annealed sample.

### Surface Area

One of the reasons why TiO_2_ NTs have been widely studied is their relatively high surface area [92]. For example, high surface area is beneficial for absorbing more dye molecules in DSSCs and, subsequently, it promotes efficiency [93]. Although the nanotubular structure contributes to a surface area that is considerably larger than that of bulk TiO_2_, there is still much room for improvement. On the one hand, much effort has been dedicated to increasing the surface area of TNTs by adjusting the NT length, diameter, and tube wall thickness. However, the average surface area of TNTs prepared by anodization and annealing processes is only approximately 30–40 m^2^ g^−1^ according to a Brunauer–Emmett–Teller (BET) analysis using N_2_ adsorption/desorption [25, 94, 95]. On the other hand, some modifications of the NT geometry, such as the fabrication of bamboo-type architecture and decoration of NTs with NPs, have been performed to improve the surface area [96–98]. Using the WAC method to decorate TiO_2_ NPs on TNTs is clearly more convenient and environmentally friendly than these methods. As shown in Table 1, some experimental parameters and their influences on TNTs properties, such as morphology and specific surface areas, are listed. As the morphology of the as-anodized NTs barely changes after annealing, the specific surface area of the annealed products is close to that of the as-anodized ones (approximately 30 m^2^ g^−1^). When the solid–liquid method is employed for crystallization, the specific surface area of the sample after water immersion remarkably increases to 104.76 m^2^ g^−1^, which is nearly four times that of the as-anodized sample. The emergence of TiO_2_ NPs makes a major contribution to the high specific surface area. Moreover, the specific surface area of a sample possessing a NR morphology (Ref. [40]) reaches as high as 203.3 m^2^ g^−1^. Although the long treatment time (72 h) plays a key role in forming a large specific surface area, the length and diameter of the original NTs are also important factors. In addition, the products prepared by the solid–gas method also exhibit a relatively high specific surface area.

**Table 1:** Experimental parameters and their influence on TNTs properties of WAC methods

Method	Temperature (°C)	Treatment time (h)	Morphology	Tube diameter (nm)	Specific surface area (m^2^ g^−1^)	References
As-anodized	25	0	NTs	80	26.67	[48]
Annealing	450	4	NTs	–	31.45	[48]
Solid–liquid	90	1	NPs/NTs	40	68.82	[48]
Solid–liquid	90	6	NPs/NTs	10	104.76	[48]
Solid–liquid	25	72	NRs	0	203.3	[40]
Solid–liquid	20	48	NPs/NTs	48	–	[47]
Solid–liquid	20	72	NRs	0	–	[47]
Solid–gas	50	2	NPs/NTs	35	–	[55]
Solid–gas	50	2	NRs	0	106	[55]
Solid–gas	180	1	NPs/NTs	20	70.8	[53]
Solid–gas	200	6	NRs	0	52.4	[43]

### Crystallinity

The amorphous TNTs can transform into the anatase phase through the dissolution–precipitation process with the assistance of water. There are two main factors influencing the crystallinity of the products: treatment duration and treatment temperature. Generally, the crystallinity is enhanced with increasing treatment time or treatment temperature; however, it should be noted that this upward trend is not unlimited. For example, a distinct (101) peak assigned to anatase TiO_2_ appeared after the sample was immersed in water for 2 days at RT. When the immersion time was prolonged to 4 days, the intensity of the (101) peak had almost no enhancement compared with that of the 2-day sample [47]. At the same time, the average crystalline size indeed decreased with the longer water treatment. Although the anatase phase can be obtained using the WAC method, the crystallinity is not as high as that of the annealed products. Fan et al. first crystallized amorphous TNTs by the solid–gas method, and then the prepared products were further annealed at 450 °C [53]. The intensity of the dominant (101) peak was significantly larger after annealing, indicating that the previously obtained TNTs were partially crystallized. As we expected, the structure and morphology were not clearly different after annealing. Therefore, some researchers first prepare rough NPs/NTs using the WAC method and then anneal them at high temperature; thus, the final products possess both high surface area and good crystallinity.

### Bandgap

As TNTs are semiconductors, their bandgap is an important characteristic that can highly influence their properties and applications. Among the normal crystalline phases of TiO_2_ (anatase, rutile, brookite), anatase TiO_2_ is widely investigated because it has a better photocatalytic activity due to its relatively low charge carrier recombination rate [99]. In a conventional manner, the amorphous as-anodized NTs are annealed to obtain anatase TiO_2_ NTs, which usually possess a bandgap of approximately 3.2 eV [100–102]. Therefore, we wonder if the bandgap differs when the anatase TiO_2_ products are fabricated by the WAC method at low temperatures.

**Fig. 12:**
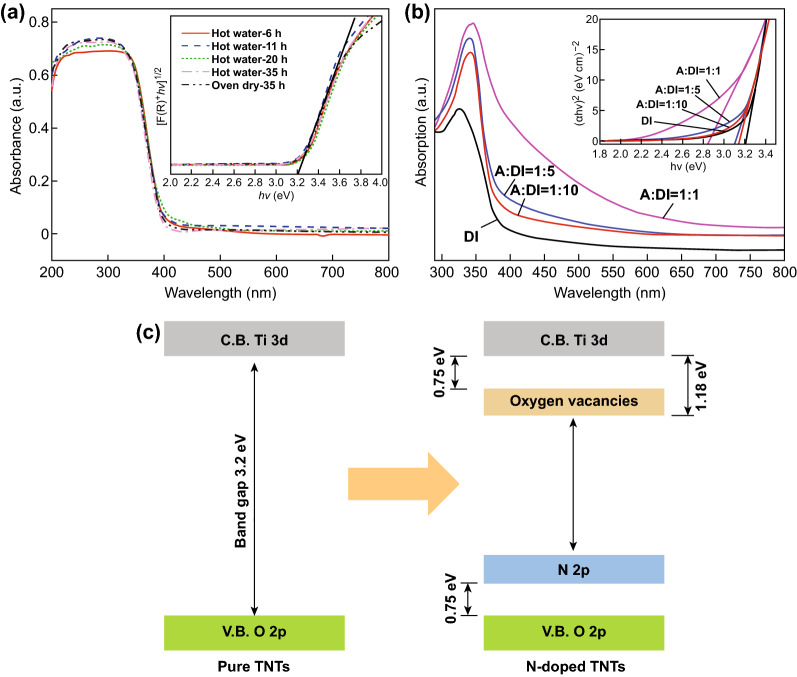
**a** UV–Vis absorption spectra of the TiO_2_ NT-powders after different heat treatment duration times at 92 °C. The inset shows the corresponding estimated bandgap. **b** The absorption spectra of water immersed TNTs and ammonia solution immersed TNTs with different concentrations. The inset shows the corresponding estimated bandgap, A/DI = Vol_ammonia_/Vol_water_. **c** Schematic of the band structure of pure TNTs and nitrogen-doped TNTs prepared by immersing the as-anodized sample in ammonia solution [39, 55]. Copyright © 2011 American Chemical Society

To assess the bandgap of the WAC-treated products, ultraviolet–visible (UV–Vis) absorption spectra of the samples were measured. Liao used a solid–liquid method to crystallize the amorphous TNTs, and Andrea used a solid–gas method. They found that the bandgap of the anatase TiO_2_ products was close to 3.2 eV (Fig. 12a) [39, 55]. Similar bandgap values indicate that the WAC-treated samples absorb similar numbers of photons as the annealed samples. However, in Andrea’s work, the TNTs prepared by the solid–gas method exhibited higher efficiency in degrading methylene blue than the annealed TNTs. As the abilities of these materials to absorb photons were very similar, this improvement could be attributed to a high surface area, as discussed above.

In addition, the bandgap changed remarkably and was no longer 3.2 eV when the amorphous TNTs were immersed in a solution containing specific ions. For example, the as-anodized TNTs were immersed in DI water and an aqueous solution of ammonia for crystallization [75]. The absorption spectra of the water-treated and the ammonia-treated samples are shown in Fig. 12b. The estimated bandgap of the water-treated sample is approximately 3.2 eV, which is in agreement with previous reports. However, all samples immersed in ammonia solution have higher absorption intensity than the water-treated samples. The bandgap decreased gradually with increasing ammonia concentration, indicating that more light was absorbed. When the ammonia: water concentration ratio was 1:1, the bandgap decreased to 2.84 eV, which could be attributed to an isolated localized state of N 2p (Fig. 12c). It is well known that the annealed anatase TNTs are only activated under UV light (wavelength < 387 nm), which is only a small fraction (4%) of the solar spectrum. Hence, the narrower bandgap (2.84 eV) leads to a broader absorption spectrum that includes visible light and, subsequently, enhances the material’s photocatalytic properties.

## Applications

### Degradation of Pollutants

**Fig. 13:**
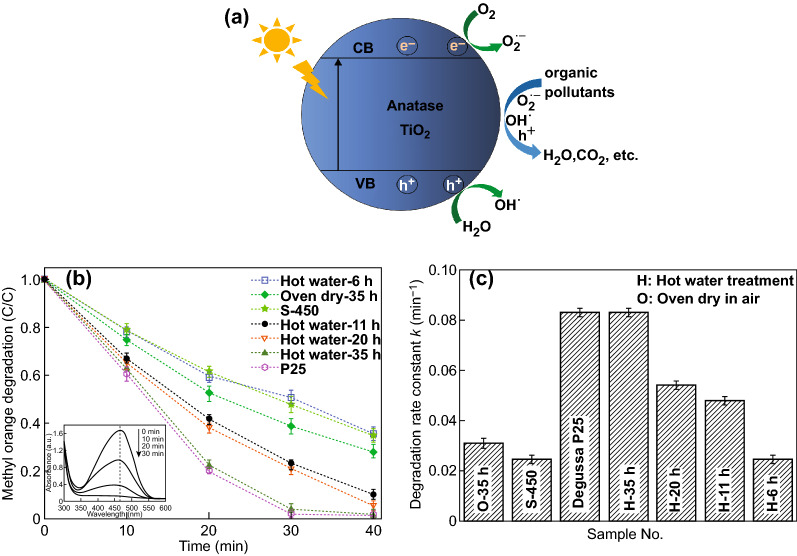
**a** Schematic illustration of the reaction process for degrading pollutants by TiO_2_ under irradiation. **b** Photocatalytic degradation kinetics of the MO (methyl orange) aqueous solution and **c** the degradation rate constant by using the TiO_2_ NT-powders obtained by water immersion with different treatment duration times at 92 °C (the inset of **b** shows the MO degradation processing with the TiO_2_ NT-powders after hot water treatment at 92 °C for 35 h as photocatalyst, and S-450 refers to the sample sintered at 450 °C for 3 h) [39]. Copyright © 2011 American Chemical Society. (Color figure online)

Recently, various photocatalytic semiconductor materials have been widely investigated [103–105]. Among them, TiO_2_ has received increasing attention because of its excellent photocatalytic performance in degrading organic pollutants [106–108]. A schematic illustration of the degradation process of TiO_2_ is shown in Fig. 13a. First, UV irradiation promotes electrons from the valence band (VB) to the conduction band (CB), and the corresponding electrons (e^−^) and holes (h^+^) reach the TiO_2_-environment interfaces. In the CB, the electrons on the surface of TiO_2_ are easily captured by oxygen dissolved in the solution, forming O2^·−^ species. O2^·−^ has a high oxidizing power and, thus, plays a vital oxidative role in the degradation process [109, 110]. At the VB, a fraction of photogenerated holes can directly oxidize the pollutants adsorbed on the surface of TiO_2_, and the other holes can react with water molecules to form the hydroxyl radical (OH^·^). Because O_2_^·−^ and OH^·^ possess high oxidative activities, the pollutants are effectively degraded to harmless substances. The related reactions are shown as follows:

6 $${\text{TiO}}_{2} + {\text{hv}} \to {\text{TiO}}_{2} \left( {{\text{e}}^{ - } + {\text{h}}^{ + } } \right)$$

7 $${\text{TiO}}_{2} \left( {{\text{e}}^{ - } } \right) + {\text{O}}_{2} \to {\text{TiO}}_{2} \left( {{\text{O}}_{2}^{ \cdot - } } \right)$$

8 $${\text{TiO}}_{2} \left( {{\text{h}}^{ + } } \right) + {\text{H}}_{2} {\text{O}} \to {\text{TiO}}_{2} \left( {{\text{OH}}^{ \cdot } } \right)$$

9 $${\text{Pollutants}} + {\text{O}}_{2}^{ \cdot - } /{\text{OH}}^{ \cdot } \to {\text{H}}_{2} {\text{O}} + {\text{CO}}_{2} + {\text{others}}$$

Liao et al. crystallized amorphous TNTs by immersing them in hot water, and their photocatalytic properties were investigated through the degradation of MO aqueous solution, as shown in Fig. 13b, c [39]. The results showed that the TNTs that received hot water treatment for 6 h exhibited a slightly higher performance than the annealed (450 °C) products. Because the crystallinity of the hot water-treated TNTs was not as high as that of the annealed samples, the approximately equal degradation efficiency was attributed to their elevated surface area. With increasing immersion time, the degradation efficiency remarkably increased. The efficiency of a 35-h-immersed sample was nearly four times that of an annealed sample. Hou et al. fabricated anatase TiO_2_ by immersing amorphous TNTs in an aqueous solution of ammonia [75]. As expected, the ammonia-treated products also presented a significant improvement over the annealed sample. Although a high surface area is an important factor that notably promotes the contact of TiO_2_ and MO aqueous solution, the narrower bandgap of the ammonia-treated products also plays a key role. Such bandgap allows the ammonia-treated products to absorb visible light more efficiently than the annealed sample and, consequently, contributes to their excellent degradation performance.

### DSSCs

In recent years, DSSCs have attracted worldwide attention as promising candidates for next-generation photovoltaics [111–113]. For conventional DSSCs, the photoanode is usually a mesoporous thin film composed of randomly distributed TiO_2_ NPs [114]. Unfortunately, the numerous boundaries between NPs cause a slow diffusion of photogenerated electrons in this system. To circumvent this issue, a variety of 1-D structures, including NTs, nanowires, and nanofibers, have been investigated to optimize the electron transport [115–117]. In particular, vertically oriented TNTs have been widely studied as an alternative because of the fast transport of electrons and ions through their TiO_2_ layers [118–120]. However, the smooth tube walls often have insufficient surface area for dye adsorption and, hence, a relatively poor light harvesting ability, which limits the improvement of conversion efficiency [121, 122]. Therefore, it is important to increase the surface area of TNTs and maintain the nanotubular structure at the same time [123, 124]. Clearly, the WAC method introduced in this review is more convenient and efficient than other methods for obtaining NPs/NTs that both possess high surface area and exhibit rapid transport. In the following section, we will introduce the basic principles of DSSCs and discuss related studies employing WAC-treated products.

**Fig. 14:**
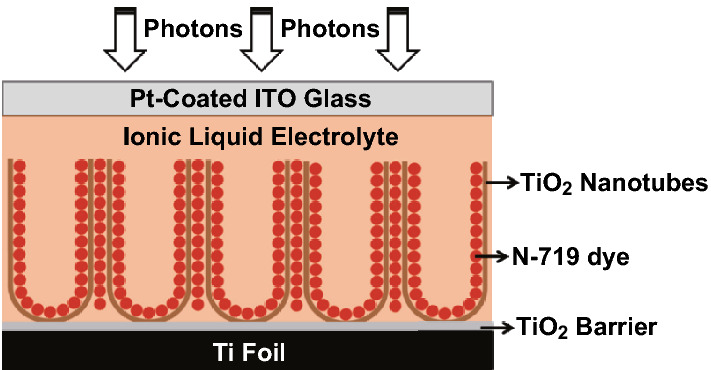
Schematic illustration of the configuration of DSSCs based on TNTs [125]. Copyright © 2012 American Chemical Society

A schematic illustration of the configuration of DSSCs is presented in Fig. 14 [125]. Incident photons are absorbed by dye molecules adsorbed on the TiO_2_ NT walls, and electrons are excited from the highest occupied molecular orbital (HOMO) to the lowest unoccupied molecular orbital (LUMO). The excited electrons are injected into the CB of TiO_2_ and then travel through the NTs via diffusion toward the back contact, finally reaching the counter electrode through the circuit. Meanwhile, the oxidized dye on the surface accepts electrons from I^−^ in the electrolyte, leading to the regeneration of the ground state of the dye and generation of I_3_^−^. Then, I_3_^−^ diffuses toward the counter electrode and reduces back to I^−^, completing the cycle [126, 127].

A high surface area and nanotubular structure are important factors that ensure that more dye molecules can be absorbed and the recombination rate is restricted. Therefore, the products synthesized by WAC are considered suitable for DSSCs. For example, as-anodized TNTs were immersed in water for several days at RT, transferred, and then applied in DSSCs [47]. After 2 days of immersion, the amount of absorbed dye increased remarkably (by 38.9%) compared with that adsorbed by the as-anodized TNTs. Correspondingly, the DSSCs based on TNTs immersed in water for 2 days exhibited the excellent solar energy conversion efficiency (*η*) of 6.06%, which is a 33% improvement compared with that of a sample employing untreated TNTs. When the immersion time was prolonged to 3 days, the dye-adsorption ability of the products increased. However, the conversion efficiency of the 3-day sample decreased to 83.3% of the 2-day sample value. Similarly, Zeng et al. fabricated NP/NT products by the solid–gas method and employed them in DSSCs [44]. The samples synthesized at 180 °C showed the highest conversion efficiency (up to 8.11%), while the efficiency of the 200 °C sample was only 6.4%. This decline was attributed to the elevated recombination rate due to the relatively collapsed nanotubular architecture, which was generated using either a long immersion time or a high temperature. We can conclude that not only a high surface area but also a good architecture is beneficial for efficiency. In Table 2, some photovoltaic parameters of DSSCs based on various photoanodes are listed. Specifically, *J*_sc_, *V*_oc_, *FF*, and *η* represent the short-circuit current density, open-circuit voltage, fill factor, and solar energy conversion efficiency, respectively. SL, SG, and MW represent the solid–liquid, solid–gas, and modified WAC methods, respectively.

**Table 2:** Photovoltaic parameters of DSSCs based on various photoanodes

Samples	*J*_sc_ (mA cm^−2^)	*V*_oc_ (V)	*FF* (%)	*η* (%)	References
SL (0 day)	9.96	0.73	63	4.57	[47]
SL (2 day)	12.67	0.73	65	6.06	[47]
SL (3 day)	10.84	0.72	65	5.07	[47]
SL (15 min)	8.82	0.70	57	3.54	[84]
SG (160 °C)	15.39	0.74	65	7.40	[44]
SG (180 °C)	16.46	0.72	68	8.11	[44]
SG (200 °C)	12.28	0.72	67	6.40	[44]
MW (24 h)	11.7	0.72	57.2	4.9	[45]
MW (48 h)	11.9	0.68	56.7	4.6	[45]
Annealed	12.04	0.71	66	5.67	[44]

### Supercapacitors

It is well known that TNT supercapacitors are a type of double-layer capacitors, where a large surface area corresponds to an excellent areal specific capacitance [128–131]. Therefore, enhancing the NT surface area is considered to be an effective approach to improve the capacitance, and numerous research efforts have been dedicated to it. Hybrid NPs/NTs possessing high surface areas prepared by the WAC method are clearly promising candidates for high-performance capacitors.

**Fig. 15:**
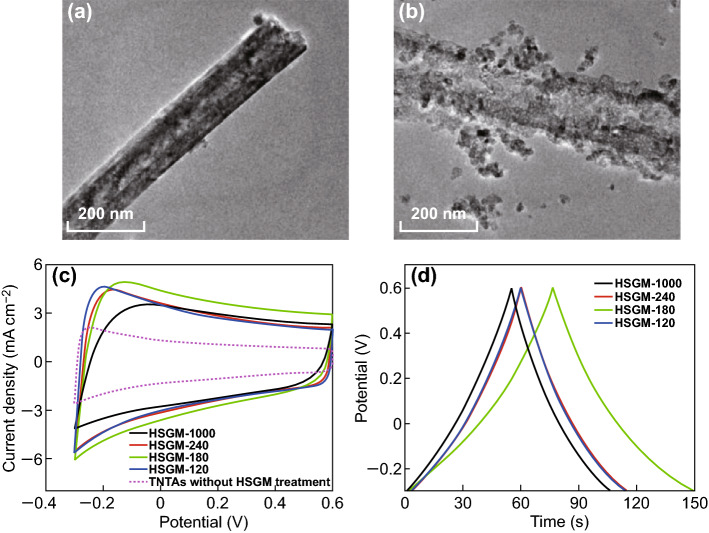
Typical TEM images of the TNTs for **a** as-anodized and **b** after HSGM (hydrothermal solid–gas method) treatment with 120 μL water put into a 50 mL Teflon autoclave. **c** Cyclic voltammograms of the TNTAs without HSGM treatment and TNTAs treated by HSGM under different vapor pressures at a scan rate of 100 mV s^−1^. **d** Galvanostatic charge–discharge (GCD) curves of the HSGM-treated TNTAs at a current density of 0.5 mA cm^−2^. HSGM-120, 180, 240, 1000 represent the different water content in the Teflon autoclave, which leads to the different vapor pressures [53]. Copyright © 2017 Elsevier B.V

Fan et al. first used the hydrothermal solid–gas (HSG) method to crystallize the as-anodized TNTs and adjust their morphology; the HSG-treated sample was then annealed to yield fully crystallized products [53]. As shown in Fig. 15a, b, the as-anodized products had smooth tube walls, while the final HSG-treated products possessed a NP/NT structure, leading to a high surface area. For the supercapacitor performance, all the HSG-treated samples exhibit larger integrated areas and higher current responses than the direct-annealed TNTs without HSG treatment, indicating a significant enhancement of capacitance by HSG treatment (Fig. 15c). From Fig. 15d, the calculated areal capacitance of the HSG-180 sample (180 μL water in a 50 mL Teflon liner) was highest, up to 41.04 mF cm^−2^, which is 2.96 times that of the direct-annealed TNTs without HSG treatment. The surface area of the HSG-180 sample is 3.16 times that of the direct-annealed TNTs without HSG treatment. The high similarity of these values suggests that the improvement in capacitance could be fully attributed to the enlarged surface area. Furthermore, when the HSG-treated sample was annealed in argon atmosphere rather than air, the areal capacitance of the HSG-180 sample further increased to 50.39 mF cm^−2^. It is believed that the oxygen vacancies (Ti^3+^) formed in argon atmosphere lead to superior electrical conductivity and thereby promote areal capacitance. In addition to using deionized water, immersing the as-anodized TNTs in the aqueous solution containing some ions is also an appealing approach. In Cui’s work, the specific capacitance of the products prepared through immersion in (NH_4_)_2_TiF_6_ solution was 2 times higher than that of a sample treated in deionized water under the same conditions [81].

In summary, WAC-treated TNTs are suitable for many applications owing to their advantages of high surface area and structures that can be easily controlled by adjusting experimental parameters, such as immersion time and treatment temperature. In addition to the applications mentioned above, TNTs are also widely used in water splitting cells, gas sensors, biomedical coatings, drug delivery, etc. [132–137]. Thus, we foresee the WAC strategy to be employed in these fields in the future.

## Other Materials

In the above sections, we have detailed the WAC strategy mainly as it regards TiO_2_ NTs. The WAC method can be used for many other materials that can be classified as TiO_2_-based materials and other metal oxides.

### TiO_2_-Based Materials

In addition to TiO_2_ NTs, there are a variety of nanostructured TiO_2_ materials, such as TiO_2_ nanospheres (TNSs) and nanofibers (TNFs). Different from the TNTs usually prepared from Ti substrate by anodization, TNSs and TNFs can be synthesized by various methods, including sol–gel, solvothermal, hydrothermal, and electrospinning methods [138–142]. Despite the large differences in synthetic methods and morphologies, the WAC method is also considered suitable for crystallization and controlling the morphology of TNSs and TNFs.

**Fig. 16:**
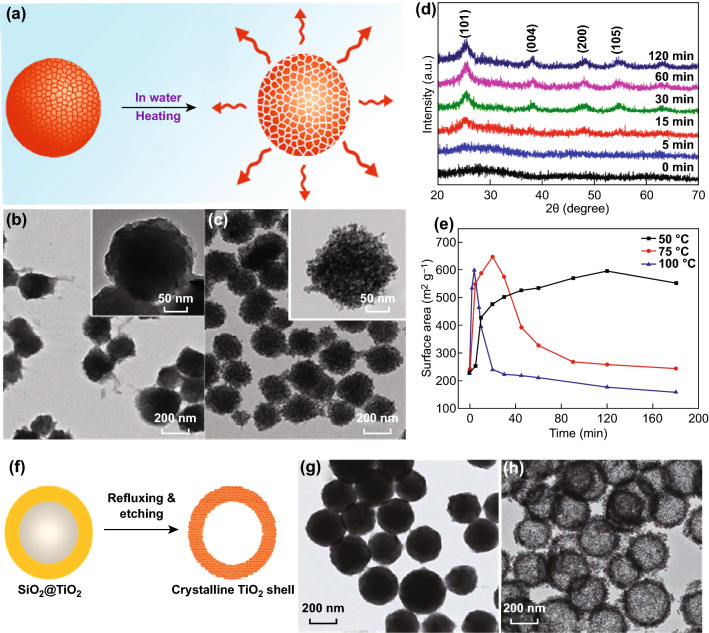
**a** Schematic illustration of the water-assisted crystallization process for preparing porous anatase TiO_2_ nanospheres from sol–gel derived amorphous particles. **b** TEM images of the original amorphous TiO_2_ spheres. **c** TEM images of the samples after immersing in water at 75 °C for 30 min. **d** XRD patterns of products obtained by immersing in water at 75 °C for different time periods. **e** The change of surface area as a function of heating time period for samples treated at different temperatures. **f** Schematic illustration of the water-assisted crystallization strategy for conversion of amorphous TiO_2_ layer to mesoporous crystalline shells. Typical TEM images of the samples at different preparation steps: **g** SiO_2_@TiO_2_ core–shell structures prepared by sol–gel coating and **h** mesoporous TiO_2_ hollow nanostructures after removing SiO_2_ cores [141, 142]. Copyright © 2011 Elsevier B.V. and 2006 American Chemical Society

*TNSs* As shown in Fig. 16a, Li et al. used the WAC method to fabricate porous anatase TNSs based on amorphous nanospheres prepared by the sol–gel technique [143]. Figure 16b shows a TEM image of pristine nanospheres just after the sol–gel process. The as-prepared nanospheres were sticky and tended to aggregate because of the presence of oligomers formed during hydrolysis. After the WAC, the original nanospheres became quite porous with the emergence of numerous NPs (Fig. 16c). When the water treatment time was prolonged, the nanospheres became more porous, and the aggregation tendency was notably relieved. In this transformation process, water mainly plays the following roles: (1) water dissolves the oligomers on the surface of as-prepared nanospheres restricting aggregation, and (2) water assists dissolution–precipitation, which eventually causes the formation of anatase grains, which are responsible for the pores of the finally obtained TNSs. The crystallinity and surface area of the TNSs were controlled by adjusting either the water treatment time or temperature. With increasing water treatment time, the crystallinity enhanced (Fig. 16d). In particular, crystalline TNSs were highly porous, reaching a surface area of as high as 647 m^2^ g^−1^ under appropriate conditions (treatment temperature: 75 °C), which is remarkably higher than the values reported in previous reports (Fig. 16e). Because of their high surface area and good crystallinity, the porous TNSs show better performance in phosphoprotein enrichment than commercial anatase products.

Joo et al. reported that porous, hollow TNSs can also be synthesized employing the WAC strategy [144]. As shown in Fig. 16f, the synthetic procedure was as follows: (1) a SiO_2_@TiO_2_ core–shell structure was first prepared by a sol–gel technique; (2) the as-prepared SiO_2_@TiO_2_ nanospheres were crystallized in water, resulting in a porous and crystalline TiO_2_ shell; and (3) the SiO_2_ core was removed, resulting in the successful fabrication of porous hollow TNSs. The corresponding morphology transformation is presented in Fig. 16g, h; it shows that porous hollow nanospheres were successfully prepared. Moreover, XRD results confirmed the transition from an amorphous form to the anatase phase after water reflux treatment. In addition, many other relevant studies have also been reported, and the WAC method is considered to be cost-effective and convenient in preparing porous TiO_2_ nanospheres [145–148].

*TNFs* TNFs, as one of the most important 1-D nanomaterials, have received extensive attention, and various methods have been developed for synthesizing them [149, 150]. Among the methods, the electrospinning technique is considered to be very promising owing to its versatility and flexibility [151–153]. Although TNFs have been investigated systematically in many ways, there are few reports about the effects of water on electrospinning TNFs. As water plays an important role in adjusting the properties of TNTs and TNSs, it can be speculated that electrospinning TNFs would also be affected by the presence of water.

**Fig. 17:**
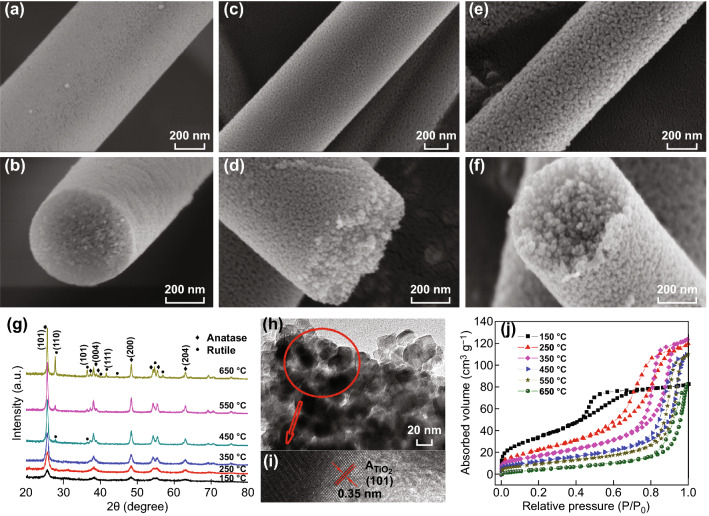
SEM images of the TiO_2_ nanofibers treated with water steam at different temperatures of **a**, **b** 150 °C; **c**, **d** 350 °C; and **e**, **f** 550 °C. **g** XRD patterns of the samples heat-treated at different temperatures in water steam. **h**, **i** HRTEM and SAED images of the water steam-treated TiO_2_ nanofibers prepared at 550 °C. **j** Nitrogen adsorption–desorption isotherms of the samples heat-treated at different temperatures in water steam [152]. Copyright © 2009 Elsevier B.V

In 2017, Jin et al. fabricated porous and crystalline TNFs by a simple water steam treatment without any template agents [154]. Similar to the previous WAC results, the morphology of the nanofibers greatly changed after water steam treatment and was highly dependent on treatment temperature. SEM images of the TNFs under water steam treatment at various temperatures are presented in Fig. 17. When the precursor nanofibers were treated at 150 °C for 2 h, the surface of the nanofibers became rough, and some pores emerged (Fig. 17a, b). With increasing temperature, the surface became rougher, and the diameter of the pores increased (Fig. 17c–f). Specifically, the average pore sizes of the 150, 350, and 550 °C samples were 3.63, 8.84, and 13.95 nm, respectively. After steam treatment, the original nanofibers became crystalline, and the crystallinity of the obtained TNFs increased with the temperature elevation (Fig. 17g), similar to the behavior of the TNTs mentioned above. Figure 17h, i presents TEM results of a sample prepared at 550 °C; they show that the rough nanofibers were composed of anatase NPs with a diameter of 30 nm. Because the vapor-treated nanofibers were composed of NPs, the porous TNFs exhibited higher surface areas than the annealing sample (Fig. 17j). The highest surface area of the water steam-treated TNFs was 128.07 m^2^ g^−1^, while that of the TNFs that were annealed in air was only 9.85 m^2^ g^−1^. This large difference can be attributed to the dissolution–precipitation process under water steam. Sun et al. also prepared TiO_2_ nanoflowers through the dissolution–precipitation mechanism [155]. It can be concluded that the WAC strategy is applicable to TiO_2_-based materials.

### Other Metal Oxides

The as-anodized amorphous TNTs can be transformed into the anatase phase with water treatment through the dissolution–precipitation process, in which the TiO_6_ octahedra rearrange and form the unit cell of anatase TiO_2_. Although this type of WAC mechanism can be extended to other TiO_2_ nanomaterials, their basic building blocks are still TiO_6_ octahedra. Therefore, we wondered whether the WAC mechanism is applicable to metal oxides that contain no TiO_6_ octahedra.

**Fig. 18:**
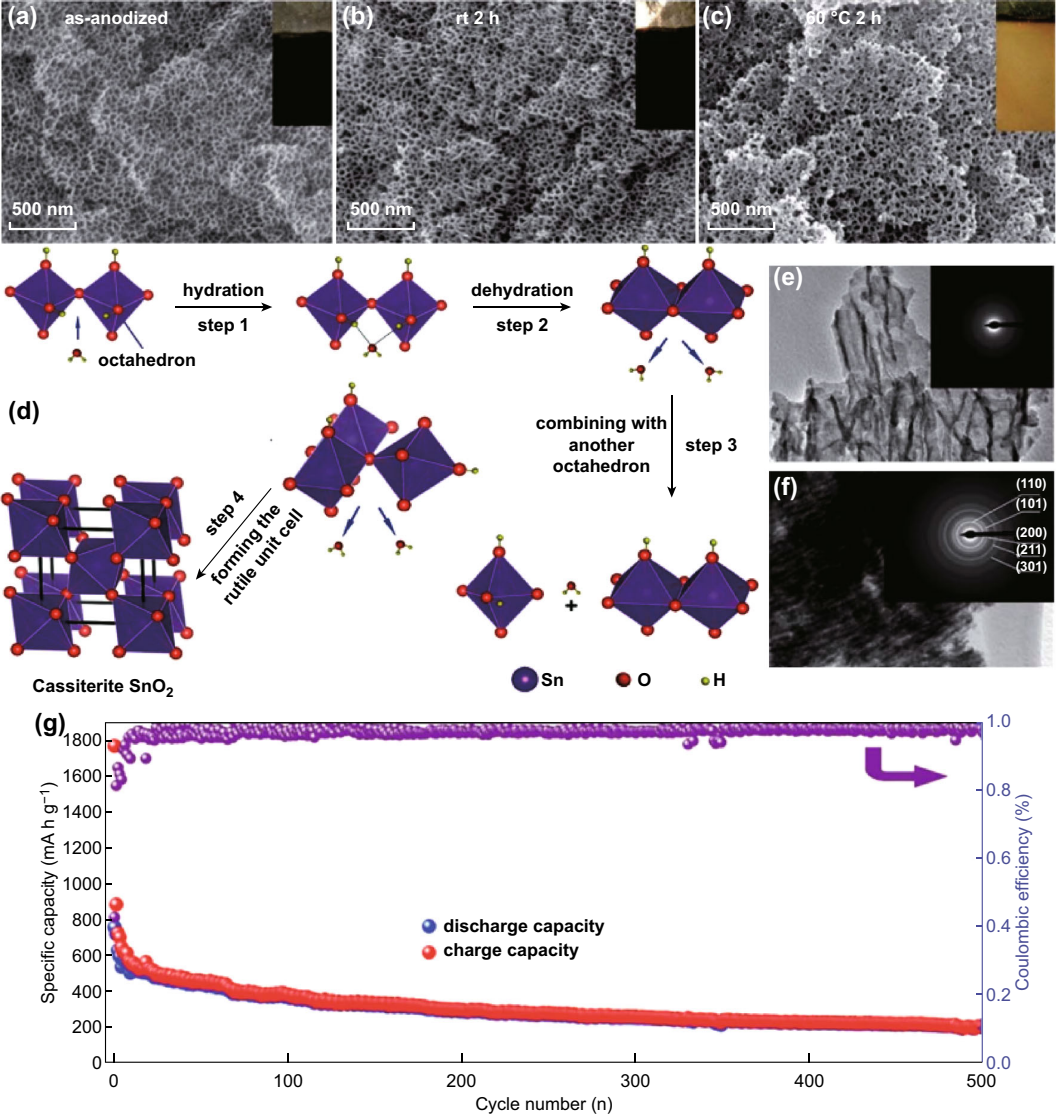
SEM images of the as-anodized SnO_2_ samples **a** before and **b** after soaking in water for 2 h at room temperature and **c** after soaking in water for 2 h at 60 °C. The insets are the photographs showing the appearance of the corresponding SnO_2_ samples. **d** Schematic illustration showing the transformation from amorphous to rutile SnO_2_ assisted by water soaking. TEM images of the SnO_2_ samples **e** as-anodized and **f** after water soaking at 60 °C for 2 h; the insets show the corresponding SAED patterns. **g** Long-term stability profiles and Coulombic efficiency of the water-crystallized SnO_2_ at 0.3 C for 500 cycles [159]. Copyright © 2015 WILEY–VCH Verlag GmbH & Co. KGaA, Weinheim

Nanostructured tin oxides (SnO_2_) synthesized by anodizing tin foils have been widely investigated in various areas [156–158]. Similar to TiO_2_, the as-anodized SnO_2_ nanomaterials are amorphous and not suitable for many applications, such as gas sensing and energy storage [159, 160]. Because the melting point of the Sn substrate is approximately 230 °C, using the conventional annealing method (500 °C) to crystallize the as-anodized SnO_2_ is impracticable. Some researchers focused on the interesting amorphous–anatase transformation of the as-anodized TiO_2_ NTs and developed a convenient way to obtain crystalline SnO_2_ at low temperatures. In 2017, Bian et al. used the WAC method to crystallize as-anodized SnO_2_ for the first time [161]. To be specific, the anodization of tin was first carried out with tin foil as the anode in an oxalic acid aqueous solution. Then, the amorphous as-anodized SnO_2_ was immersed in deionized water at various temperatures (25, 40, 80, or 100 °C) for different durations. Although no clear change was observed when the as-anodized sample was immersed at 25 °C for the short time of 2 h (Fig. 18a, b), prolonging the immersion time to 168 h effectively crystallized the amorphous sample. In addition, the higher treatment temperature accelerated the crystallization. Both digital photographs and SEM images of the products prepared at 60 °C are quite different from those of the as-anodized and RT-treated samples. As the building blocks of SnO_2_ (amorphous and rutile) are SnO_6_ octahedra, similar to TiO_6_ octahedra in TiO_2_, the Bian group proposed a mechanism explaining that the transformation from amorphous to rutile SnO_2_ was assisted by water soaking (Fig. 18c). This mechanism also includes a hydration-dehydration process similar to that of TiO_2_, as discussed in Fig. 3c. As shown in Fig. 18e, TEM and SAED images reveal the porous structure and amorphous nature of the as-anodized SnO_2_. After the samples were soaked in water at 60 °C for 2 h, clear and strong diffraction rings assigned to rutile SnO_2_ appear (Fig. 18f), indicating successful amorphous–crystalline transformation with the assistance of water. The WAC-treated SnO_2_ products were applied in sodium-ion storage and delivered excellent performance, as shown in Fig. 18g.

In addition, the WAC method was also proven to be applicable to the as-anodized iron oxides in our previous work [162]. The facile WAC method is appropriate for the crystallization of not only TiO_2_ NTs but also many other metal oxides at low temperatures.

## Conclusion and Outlook

The WAC strategy has been extensively studied and it is considered to be an efficient and convenient approach to crystallizing amorphous TiO_2_ NTs at low temperatures. In this review, we have summarized various aspects of the recent progress in using this strategy. The basic WAC method is simply immersing as-anodized TNTs in water, during which the disordered TiO_6_ octahedra are rearranged with the assistance of water. Although many modified methods have been developed, water molecules still play the most important role in causing the amorphous-anatase transformation. In addition, the WAC strategy can also be employed for doping metal or nonmetal elements into TNTs by using solutions containing different ions. Because of the dissolution–precipitation process, some crystalline TiO_2_ NPs emerge on the tube walls, leaving a rough surface feature, which is quite different from the tube walls of the annealed products. Because of the numerous NPs, the WAC-treated products usually possess a high surface area. Furthermore, we can control the morphology of the samples by adjusting experimental parameters, such as the immersion time and treatment temperature. Combining the merits of 1-D architecture and high surface area, the WAC-treated products show excellent performance in many applications, including photocatalysis, DSSCs, and supercapacitors. It should be noted that the WAC mechanism is not only applicable to amorphous as-anodized TNTs; it was proven that many other TiO_2_ nanomaterials prepared by various techniques can also be crystallized through this type of WAC method.

Despite many clear advantages of the WAC strategy for crystallization, there are still some drawbacks, such as the relatively long crystallization time and low degree of crystallinity. Therefore, optimizing the WAC method to overcome these challenges requires further study. Although we have shown that the WAC strategy is also applicable to as-anodized tin or iron oxides, there are still many metal oxides that have not been investigated in this sense. Hence, another topic that must be further studied is the expansion of the WAC method to other metal oxides. We hope that this review will motivate the development of WAC strategies in more fields and inspire researchers in low-temperature crystallization.
